# Focused Ultrasound-Induced Blood-Brain Barrier Opening Enhances GSK-3 Inhibitor Delivery for Amyloid-Beta Plaque Reduction

**DOI:** 10.1038/s41598-018-31071-8

**Published:** 2018-08-27

**Authors:** Po-Hung Hsu, Ya-Tin Lin, Yi-Hsiu Chung, Kun-Ju Lin, Liang-Yo Yang, Tzu-Chen Yen, Hao-Li Liu

**Affiliations:** 1 Center for Advanced Molecular Imaging and Translation, Chang Gung Memorial Hospital, Taoyuan, Taiwan; 2 Department of Electrical Engineering, Chang Gung University, Taoyuan, Taiwan; 3 Graduate Institute of Biomedical Sciences, Department of Physiology and Pharmacology, Chang Gung University, Taoyuan, Taiwan; 4 Department of Nuclear Medicine, Chang Gung Memorial Hospital, Taoyuan, Taiwan; 5 Department of Physiology, School of Medicine, College of Medicine, China Medical University, Taichung, Taiwan; 6 Department of Biotechnology, College of Medical and Health Science, Asia University, Taichung, Taiwan; 7 Department of Neurosurgery, Chang Gung Memorial Hospital, Taoyuan, Taiwan

## Abstract

Alzheimer’s disease (AD) is a neurodegenerative disease that is the leading cause of age-related dementia. Currently, therapeutic agent delivery to the CNS is a valued approach for AD therapy. Unfortunately, the CNS penetration is greatly hampered by the blood-brain barrier (BBB). Focused-ultrasound (FUS) has been demonstrated to temporally open the BBB, thus promoting therapeutic agent delivery to the CNS. Recently, the BBB opening procedure was further reported to clear the deposited Aβ plaque due to microglia activation. In this study, we aimed to evaluate whether the use of FUS-induced BBB opening to enhance GSK-3 inhibitor delivery, which would bring additive effect of Aβ plaque clearance by FUS with the reduction of Aβ plaque synthesis by GSK-3 inhibitor in an AD mice model. FUS-induced BBB opening on APPswe/PSEN1-dE9 transgenic mice was performed unilaterally, with the contralateral hemisphere serving as a reference. GSK-3 level was confirmed by immunohistochemistry (IHC) and autoradiography (ARG) was also conducted to quantitatively confirm the Aβ plaque reduction. Results from IHC showed GSK-3 inhibitor effectively reduced GSK-3 activity up to 61.3% with the addition of FUS-BBB opening and confirming the proposed therapeutic route. ARG also showed significant Aβ-plaque reduction up to 31.5%. This study reveals the therapeutic potentials of ultrasound to AD treatment, and may provide a useful strategy for neurodegenerative disease treatment.

## Introduction

Alzheimer’s disease (AD) is a degenerative brain disease and the most common cause of age-related dementia. An estimated 5.4 million Americans have Alzheimer’s disease and by mid-century the number is projected to grow to 13.8 million in the United States^1^. As the foremost threat to healthy aging, it is anticipated that AD will overwhelm the future health care system. Finding an effective treatment is thus of paramount importance. It is well known that patients with AD have a progressive deposition of amyloid-β (Aβ) plaques and neurofibrillary tangles in the brain. Aβ has long been recognized as a critical indicator of AD progression. In AD brains, Aβ peptides aggregate and form extracellular plaques. Animal studies delivering anti-Aβ antibodies directly to the cortex have demonstrated a rapid therapeutic response, but this approach requires an invasive surgical technique^2^. Data collected from cerebrospinal fluid (CSF) biomarkers and positron emission tomography (PET) imaging from AD patients indicate that Aβ is the first biomarker to accumulate, followed by the appearance of synaptic dysfunction and increased tau-protein concentrations^3^. The design of novel therapeutic agents such as monoclonal antibodies (mAbs), stem cells and genes is currently under way and clinical trials have been attempted, however, the therapeutic efficacy has been greatly limited by the fact that large molecular agents cannot penetrate the blood-brain barrier (BBB).

Low-pressure burst-mode transcranial focused ultrasound (FUS) exposure with the presence of microbubbles can locally, temporally and reversibly open the BBB^4^. BBB opening can increase the local concentration of therapeutic agents in the brain without damaging normal tissue, and via FUS-BBB opening to enhance CNS penetration of AD-treated therapeutic agents is an attractive approach. It was previously reported that targeted FUS-induced BBB opening facilitates CNS penetration of systemically administered anti-Aβ antibodies to targeted brain regions in the TgCRND8 and APP23 mouse model of AD, therefore reducing plaque load^5^, therefore confirms the feasibility of this strategy in promoting behavioral and memory recovery in a transgenic animal model^6,7^. Two potential routes for FUS-mediated plaque reduction been previously reported: (1) FUS-induced BBB opening delivered the endogenous IgG and IgM from the periphery into the brain and contributes to plaque clearance^8^. (2) Mild immune responses are activated by FUS, microglia was activated to internalize amyloid and contribute to plaque reduction^7,8^. Although the accurate mechanism of Aβ and Alzheimer’s disease is not fully explored yet, plaque reduction still serve as a potent strategy to treat AD.

Alzheimer’s disease is also characterized by the presence of Amyloid- β plaques which were composed primarily of 40- and 42-amino acid peptides—Aβ_40_ and Aβ_42_, respectively—derived from amyloid precursor protein (APP). Recent research on glycogen synthase kinase 3 (GSK-3) revealed that elevated GSK-3 activity is directly linked to increased levels of Aβ production and Aβ deposits in AD patients and AD animal models^9^. GSK-3 served as a primary kinase to be responsible for Aβ production^10,11^, Tau phosphorylation^12^ and neuroinflammation^13^. Deregulation of GSK-3 activity in neurons has been postulated as a key feature in Alzheimer’s disease pathogenesis. This is based on the interaction of GSK-3 (and more precisely, its beta isoform, GSK-3β) with many of the cellular components related to the neuropathology of AD, such as the amyloid precursor protein, the Aβ peptide, the metabolic pathway leading to acetylcholine synthesis, which are mutated in many cases of familial AD. Based on reported multiple cellular and pathological changes, inhibition of GSK-3 activity has been used to test agents and drugs on experimental rodent models, and on AD patients^9,14^. Since GSK-3 may be a potential therapeutic target for AD, a considerable amount of effort has been directed into the discovery and development of GSK-3 inhibitors in recent years^15^. At present, several chemically diverse families have emerged as GSK-3 inhibitors^16^, including peptides^17^, metal ions^18^, and diverse small heterocycles^19^, and the development of GSK-3 inhibitor recently became a potential therapy for AD. AR-A014418 is a known small-molecule GSK-3 inhibitor, which has been shown to restrain the GSK-3 activity and further decrease the phosphorylation of Tau protein at sites Ser-396/404^20^. Therefore, we hypothesized that enhanced GSK-3 inhibitor delivery via FUS-induced BBB opening might further increase AD therapeutic efficacy.

**Figure 1:**
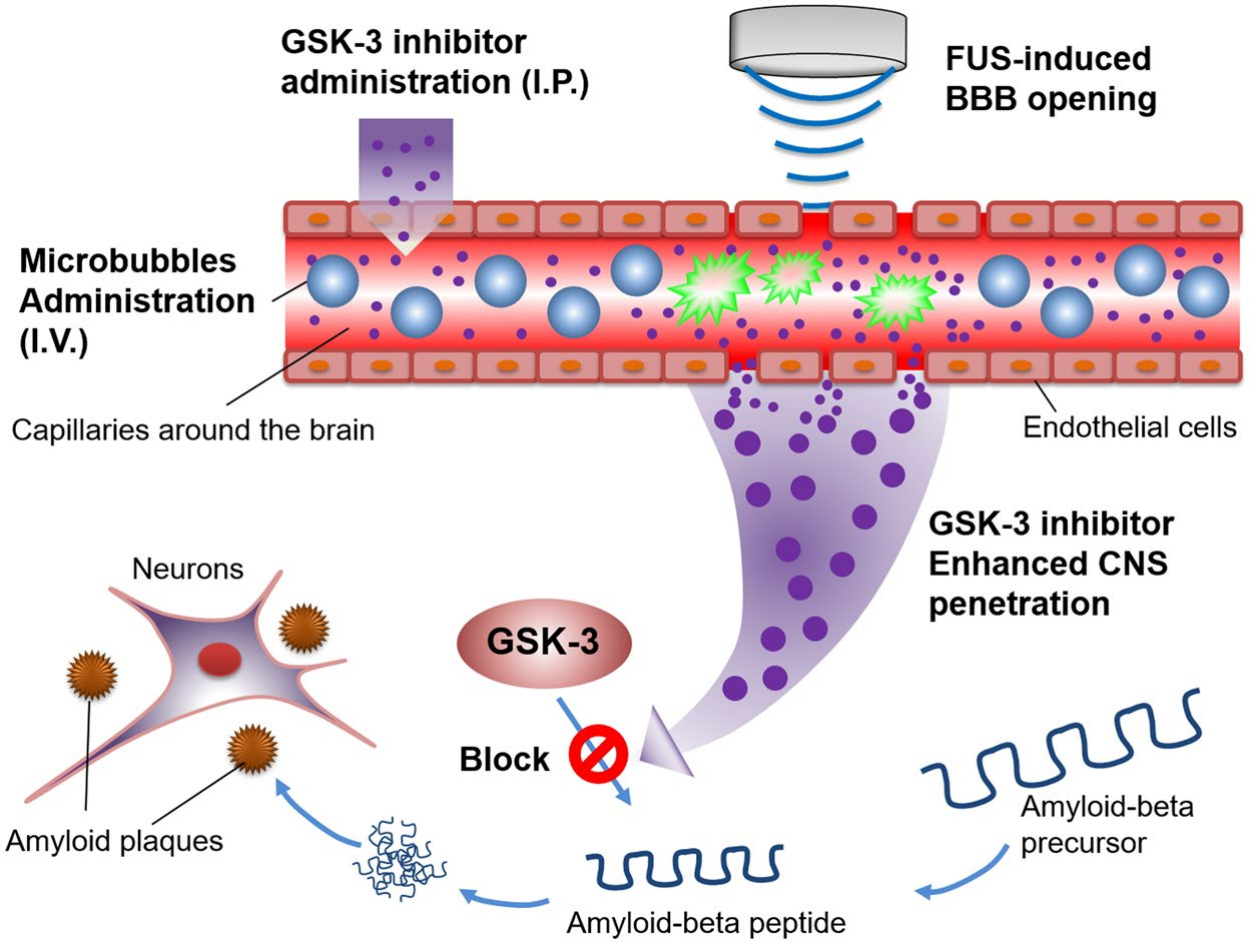
The conceptual schematics of this study. The GSK-3 inhibitor was intraperitoneally (i.p.) injected and microbubbles were intravenously (i.v.) injected. During the sonication induced by a focused ultrasound transducer, GSK-3 inhibitor penetrated the blood-brain barrier to block/reduce the Aβ peptide synthesis and overexpression.

In this study, we aimed to evaluate whether the use of FUS exposure to enhance GSK-3 inhibitor (AR-A014418) delivery can trigger the down regulation of Aβ synthesis and overexpression (concept see Fig. 1). To validate the treatment efficiency, radioactive examination and histological analysis were applied to support observation of Aβ plaque deposition changes and the proposed therapeutic route of the FUS-induced GSK-3 inhibitor enhanced delivered procedure.

## Results

### Level of GSK-3 with response to AR-A014418 treatment

**Figure 2:**
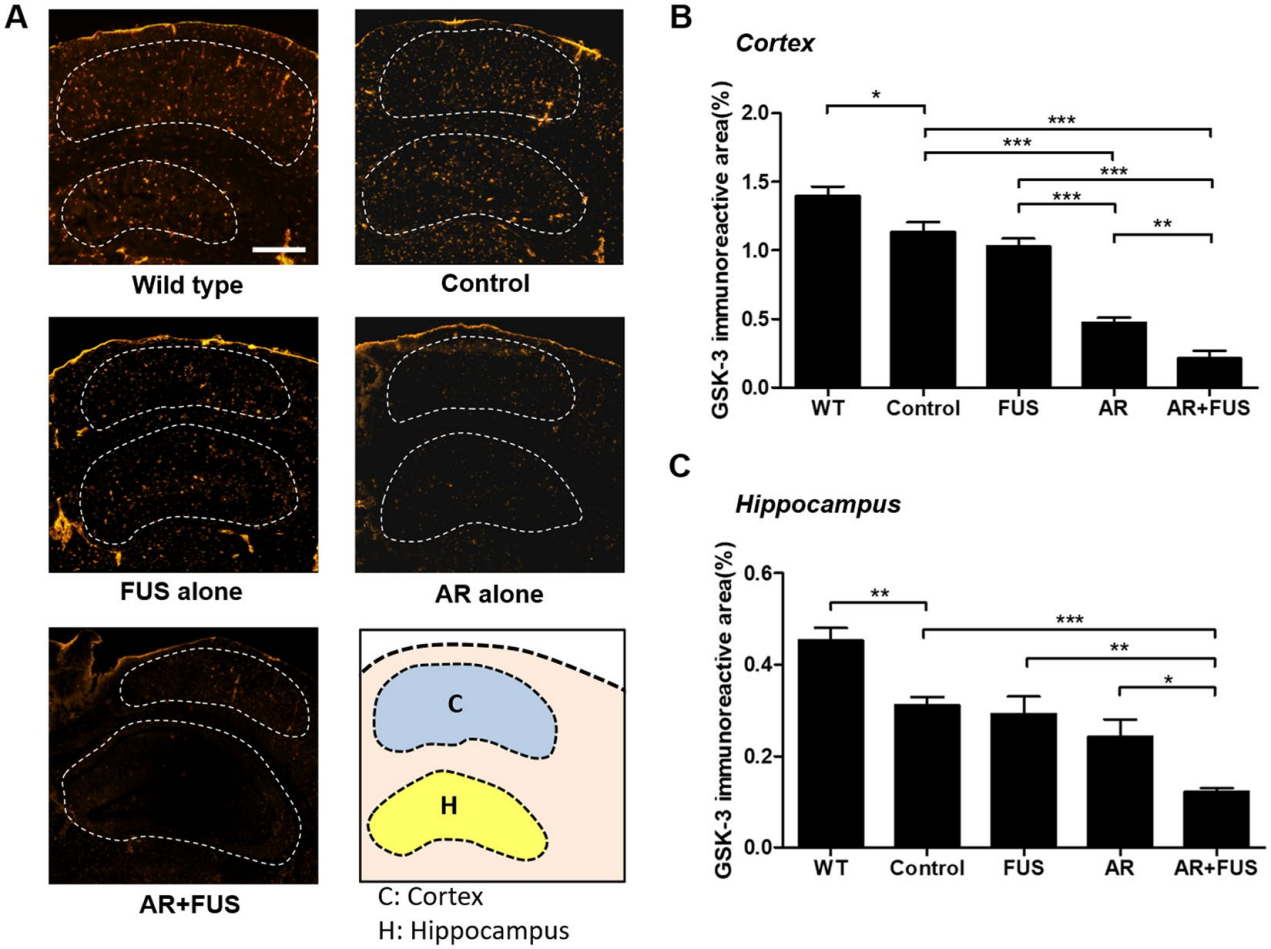
Assessment of GSK-3 distribution under various FUS and GSK-3 inhibitor treatment combinations. (**A**) Representative GSK-staining observed from wild type group (n = 3), control group (n = 3), FUS alone group (n = 6), GSK-3 inhibitor (AR) alone group (n = 6), combined treatment group (n = 9). (**B**) Quantitative analysis among groups in cortex (represented as the ratio of the GSK-3 immunoreactive area to the selected cortex region). Relative distribution was analyzed to 1.4 ± 0.07%, 1.14 ± 0.07%, 1.03 ± 0.06%, 0.48 ± 0.03%, and 0.21 ± 0.06% in wild type, control, FUS-alone, AR-alone, and AR + FUS group, respectively. AR alone groups showed significant inhibitions between control group (p < 0.001) and FUS-alone group (p < 0.001), whereas AR + FUS groups also showed significant differences between control group (p < 0.001), FUS-alone group (p < 0.001), and AR-alone group (p < 0.01). Control group showed a significant difference between the wild type group (p < 0.05). (**C**) Quantitative analysis among groups in hippocampus. The ratio of GSK-3 immunoreactive area to the whole hippocampus region showing the relative distribution of 0.45 ± 0.03%, 0.31 ± 0.02%, 0.29 ± 0.04%, 0.24 ± 0.04%, and 0.12 ± 0.01% in wild type, control, FUS-alone, AR-alone, and AR + FUS group, respectively. AR + FUS group showed significant differences between control group (p < 0.001), FUS alone (p < 0.01) as well as AR alone group (p < 0.05). Control group also showed a significant difference compared to the wild type group (p < 0.01). AR = AR-A014418; FUS = focused ultrasound. Scale bar = 500 μm.

GSK-3 staining were performed to confirm and quantitate GSK distribution to support the proposed hypothesis that enhancing GSK-3 inhibitor delivery contributes to the inhibition of Aβ plaque synthesis. Figure 2 shows the typical GSK-3 immuno-reactivity staining among the five groups, whereas the orange spots show the GSK-3 distribution at cortex and hippocampus areas (Fig. 2A). Active GSK-3 production was demonstrated in AD mice. FUS-BBB opening alone did not have a noticeable effect on GSK-3 activity in cortex nor hippocampus compared to the control group. As a contrast, inhibitor administration alone regulated GSK-3 activity effectively in cortex and slightly in hippocampus (Fig. 2B,C), thus validating the therapeutic pathway of AR-A014418. Combined FUS-BBB opening with inhibitor delivery showed the most significant reduction in GSK-3 distribution from the gross GSK-stained observation, indicating that the FUS-BBB primarily enhances inhibitor delivery into the brain for GSK-3 down regulation.

The quantitative analysis of GSK-3 were shown in Fig. 2B (cortex) and Fig. 2C (hippocampus). In AR-alone group, GSK-3 distribution in cortex was significantly down-regulated comparing to the control group (58.2%, p = 0.0002), but showed no difference in hippocampus (p = 0.1654). However, for the brain hemisphere that was exposed to FUS combined with inhibitor treatment, the most profound GSK-3 down-regulation was observed when comparing with the control mice (0.21 ± 0.06% vs. 1.14 ± 0.07%, equivalent to 81.6% reduction in cortex; 0.12 ± 0.01% vs. 0.31 ± 0.02%, equivalent to 61.3% reduction in hippocampus), and the effect was statistically significant (p < 0.0001 in cortex and hippocampus). It was also noted that the GSK-3 down-regulation effect was improved approximately 50–56% when additional applying with FUS-BBB opening efficiency after GSK-3 inhibitor administration (0.48 ± 0.03% vs. 0.21 ± 0.06%, p < 0.01 in cortex; 0.24 ± 0.04% vs. 0.12 ± 0.01%, p = 0.0196 in hippocampus). The wild type groups showed significant differences (1.4 ± 0.07% vs. 1.14 ± 0.07%, p < 0.05 in cortex; 0.45 ± 0.03% vs. 0.31 ± 0.02%, p < 0.01 in hippocampus) when compared to the control groups.

In addition, the level of inactivated form of GSK-3β (phosphorylated GSK-3β) in transgenic mice were also confirmed by western immunoblot and the results shown significantly increase in the combination of FUS and AR treatment comparing to AR alone group (Fig. S3).

### Quantification of amyloid-β plaques by autoradiography (ARG)

**Figure 3:**
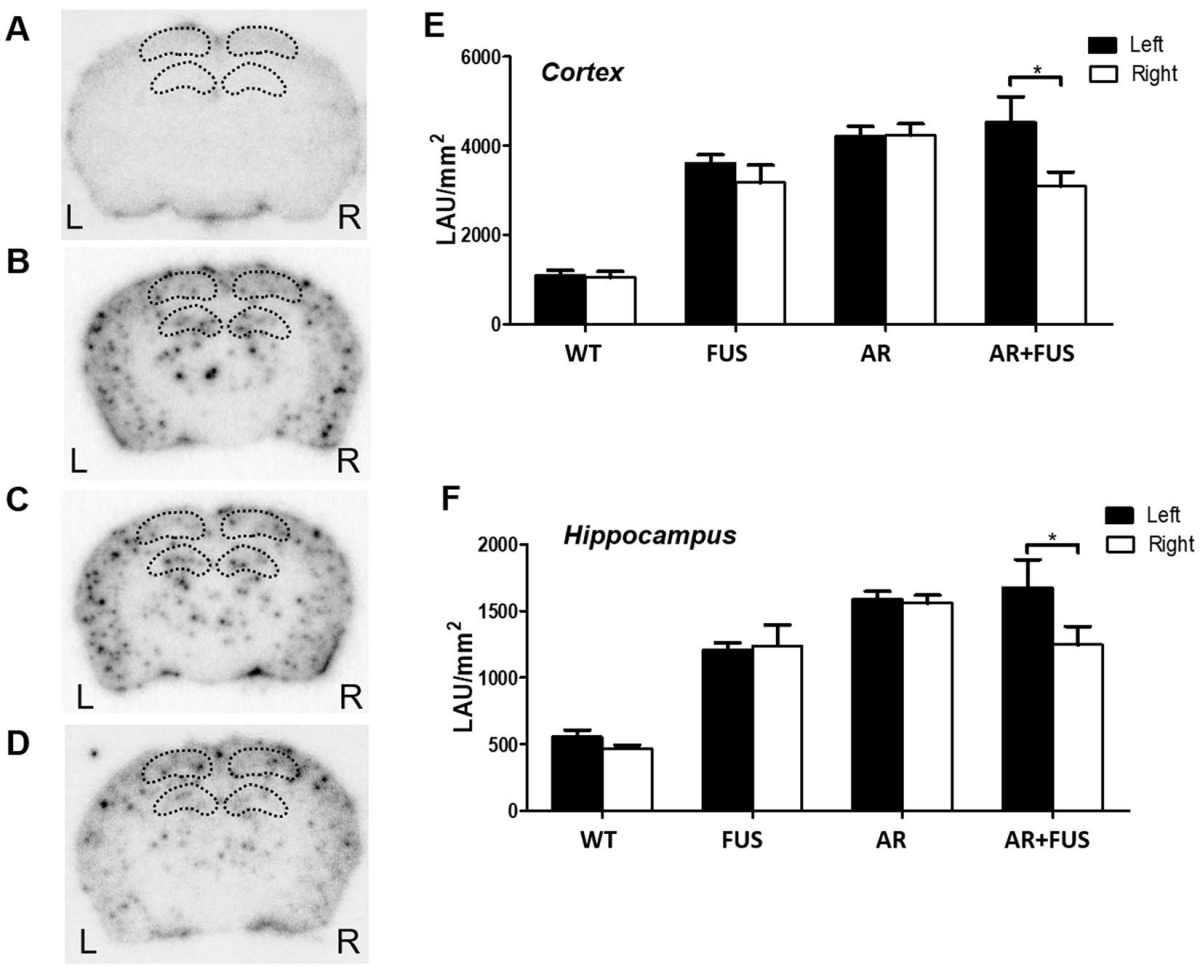
Autoradiogram of brain section images for the different treatment groups. (**A**) GSK-3 inhibitor alone with wild type mice (n = 3); (**B**) FUS sonication alone with transgenic mice (n = 6); (**C**) GSK-3 inhibitor with transgenic mice (n = 6); (**D**) combination of GSK-3 inhibitor and FUS with transgenic mice (n = 9); and quantification of 4 groups with (**E**) cortex (**F**) hippocampus. AR-alone and FUS-alone groups didn’t show plaque differences when analyzing cortex and hippocampus regions. However, the plaque-reduction effect could be revealed in AR + FUS group as measured in cortex (31.5% reduction; 3099.9 ± 311.6 LAU/mm^2^ vs. 4526.2 ± 574.9 LAU/mm^2^; p = 0.0401) or hippocampus (25.6% reduction; 1248.1 ± 136.9 LAU/mm^2^ vs. 1678.1 ± 207.1 LAU/mm^2^; p = 0.0159). WT = wild type; AR = AR-A014418; FUS = focused ultrasound.

Radiolabeled AV-45 tracer was employed to image β amyloid depositions *in vivo* via microPET, yet no significant difference of AV-45 uptake between the FUS-absent and FUS-BBB opened hemisphere (see Fig. S4). Since AV-45-tagged plaque detectability can be further improved through radiographic examination, we determined to conduct ARG brain observations for all four experimental groups (Fig. 3A–D; wild-type control, FUS alone, AR (i.e., GSK-3 inhibitor) alone and the combined AR/FUS; black dots indicated radio-labelled AV-45 attached to amyloid-beta plaques in brains). The amount of plaques was calculated separately in two different ROI definitions: cortex and hippocampus; the quantitative comparisons are shown in Fig. 3E,F, respectively. The quantitative results were presented in LAU/mm^2^, which means relative count intensity in specific area. Transgenic APPswe/PSEN1-dE9 mice spontaneously deposited more abnormal plaques than the wild-type ones, and the plaque reduction effect was not apparent when conducting sequential inhibitor administration. In AR alone group, there was no obvious difference between bilateral brain regions. Hemisphere that underwent FUS alone (i.e., FUS group) neither showed statistically sufficient plaque differences when comparing to cortex nor hippocampus. However, the plaque-reduction effect could be enhanced when combining the FUS-BBB opening with inhibitor administration (i.e., AR + FUS group) as measured when measured in the cortex (31.5% reduction; 3099.9 ± 311.6 LAU/mm^2^ vs. 4526.2 ± 574.9 LAU/mm^2^; p = 0.0401) or hippocampus (25.6% reduction; 1248.1 ± 136.9 LAU/mm^2^ vs. 1678.1 ± 207.1 LAU/mm^2^; p = 0.0159).

### Assessment of amyloid-β plaques reduction by immunofluorescence (IF)

**Figure 4:**
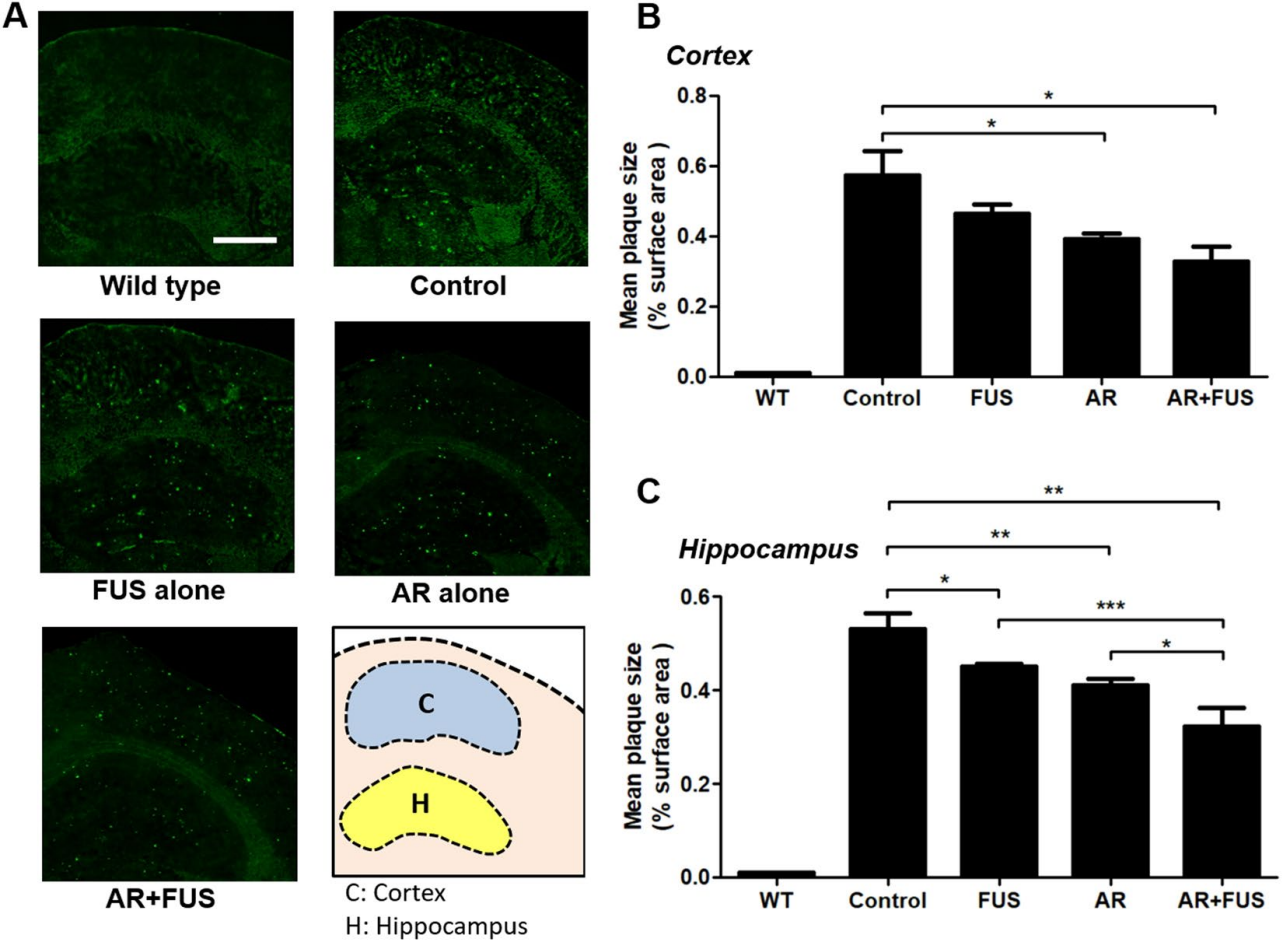
Assessment of amyloid-β plaques by fluorescent staining. (**A**) Representative Thioflavin-S stains among groups. (**B**) Quantification of amyloid-β plaque sizes in the cortex and (**C**) hippocampus, the data are represented as the ratio of the green fluorescence area from the region-of-interest (ROI). FUS alone only contribute a significant difference in hippocampus region when compared to the control group (15.1% reduction compared to control, p < 0.05). When GSK-3 inhibitor was administrated alone, significant reductions of amyloid-β plaque can be observed in both regions compared to the control group (31.6% and 22.6% reduction compared to control, with p < 0.05 and p < 0.01 in cortex and hippocampus respectively). When GSK-3 inhibitor was mediated by using FUS, there were reductive effects compared to respective control group (42.1% and 39.6% reduction compared to control, with p < 0.05 and p < 0.01 in cortex and hippocampus respectively). It was noted that combined AR + FUS treatment also induced the additive plaque reduction effect when compared to FUS alone (28.9%, p = 0.0003) and inhibitor alone (22%, p = 0.0236) in hippocampus when compared to control. AR = AR-A014418; FUS = focused ultrasound. Scale bar = 500 μm.

In addition to quantitative analysis of AV-45 autoradiograms, we performed thioflavin-S fluorescent staining to confirm the plaque distribution in brain sections. Figure 4A shows the typical fluorescent microscopic observations in the five groups (wild-type, control, FUS alone, AR alone, and combined AR/FUS treatment; the detected plaques could be visualized with green fluorescence). Figure 4B,C showed the corresponding fluorescent microscopy to observe Thioflavin-S tagged plaque in cortex and hippocampus respectively. Compared to AD mouse brain hemisphere without any intervention (control group, neither inhibitor nor FUS exposure; 0.57 ± 0.07% in cortex and 0.53 ± 0.04% in hippocampus), statistically significant plaque reduction was observed in hippocampus when comparing with the FUS alone treatment (19.3% reduction to 0.46 ± 0.03%, p = 0.2536 in cortex; 15.1% reduction to 0.45 ± 0.01%, p = 0.0312 in hippocampus). GSK-3 inhibitor administration alone provided superior plaque reduction effect both in cortex and hippocampus than FUS exposure and can reach statistical difference (31.6% reduction to 0.39 ± 0.02%, p = 0.0446 in cortex; 22.6% reduction to 0.41 ± 0.01%, p = 0.0090 in hippocampus). However, combined GSK-3 inhibitor and FUS-BBB opening provided the most plaque-reduction effect (42.1% reduction to 0.33 ± 0.04%; p = 0.0121 in cortex; 39.6% reduction to 0.32 ± 0.04%; p = 0.0096 in hippocampus). It was also noted that combined AR + FUS treatment further reduced 22% when comparing to AR alone group (p = 0.0236) and 28.9% when comparing to FUS alone group (p = 0.0003). It was noted that combined AR + FUS treatment also induced the effect when compared to FUS alone (28.9%, p = 0.0003) and inhibitor alone (22%, p = 0.0236) in hippocampus. The above results demonstrated from AV-45 radiogram of brain sections and Thioflavin-S fluorescent microscopy of brain sections, it may be concluded that inhibitor alone or FUS alone both produced plaque reduction effect, but the combination of FUS with inhibitor therapy can provide the most significant plaque-reducing effect.

## Discussion

In this study, we demonstrated that focused ultrasound (FUS)-induced BBB opening to enhance GSK-3 inhibitor delivery can efficiently reduce amyloid-beta plaques in transgenic mouse brains. GSK-3 was confirmed to play the role to down-regulate the synthesis and formation of Aβ plaques, and the proposed scheme effectively reduced GSK-3 distribution up to 81.6% (see Fig. 2B). We also showed that the use of AV-45 PET imaging has potential as diagnostic tool to *in vivo* quantitate plaque deposition (Fig. S4), while autoradiography (ARG) can provide radiolabeled tracer detectability. The reduction in Aβ plaques in the hippocampus and cortex was significant (25.6% and 31.5%, respectively when compared to control, see Fig. 3E,F). Since FUS-BBB opening alone have previously proofed its usefulness in removing existing plaques due to microglia/immunogenic activation, adding GSK-3 inhibitor enhanced delivery to regulate plaque synthesis together provides an additive strategy to further reduce the plaque deposition. This study reveals the therapeutic potentials of ultrasound to AD treatment, and may provide an alternative for neurodegenerative disease treatment.

GSK-3 plays important roles in various tissues, and its peripheral effect should be considered before applying GSK-3 inhibitors for AD treatments clinically. Previously, it has been reported that GSK-3 involve in the glycogen synthase regulation primarily in the muscle after insulin signaling, especially in those pathologies where an over-expression of the AD-related enzyme^21^. In addition, McManus *et al*. reported that the muscle glycogen levels when inhibiting GSK-3 did not cause major fluctuation when comparing to control in GSK-3 knock-in animals^22^. It therefore suggests that peripheral concerns when inhibiting GSK-3 might be manageable, owing to the additional benefit that FUS-BBB opening in promoting localized CNS GSK-3 inhibitor deposition and hence reduce systemic retention.

Previous studies have demonstrated that FUS-induced BBB opening may trigger CNS immune-regulation to facilitate Aβ clearance^7,8^ and animal behavioral functions can be restored^6,7^. Yet, these studies only demonstrated the usefulness in reducing Aβ plaques that were already synthesized and folded, and unable to interfere in upstream pathway control or even blockage. In this study, we observed that FUS-induced BBB opening alone can trigger Aβ plaque reduction (see Figs 3 and 4), which was consistent with findings from previous reports. Yet, we noted that FUS-induced BBB opening combined with GSK-3 inhibitor delivery had an additive effect on plaques reduction efficiency reaching 39.6% reduction, compared to 15.1% with FUS-BBB opening alone and 22.6% with GSK-3 inhibitor administration alone (see Fig. 4B,C). In addition, Burgess *et al*. demonstrated a plaque reduction that effectively reached 20% and showed an associated improvement in cognitive performance in the Y maze test^6^. Although not tested, we assume that animal behavior including memory and cognitive function might be significantly improved by the plaque reduction found in this study (39.6% reduction in plaque size in this study).

In the report of Burgess *et al*., Aβ plaques were observed by 3 months in the animal model (TgCRND8) and applied a 3-week FUS procedure (once per week) at the age of 7 months. In Leinenga *et al*., Aβ plaques were observed in APP23 transgenic animal model around 6 months and FUS treatments were applied for 6 weeks around 16.8 months. In contrast, plaques appeared around 6-month-old mice in APPswe/PSEN1-de9 mice employed in this study, which is similar to the APP23 model. The amyloid plaque was both concluded in these two studies despite the model difference and showed behavioral improvement. Besides, the activation of microglia was considered as the major mechanism to assist plaque clearance in the studies of Leinenga *et al*. and Jordao *et al*.^7,8^ These reports post strong implication that the proposed mechanism (microglia activation) is convincing and should be independent to the animal model.

In this study, we employed FUS to induce localized BBB-opening in single hemisphere with an exposure frequency of 7 days/exposure for a total of 5 times with the purpose of testing regional plaque reduction. Burgess *et al*. attempted to open the bilateral hippocampus (with an exposure frequency of 7 days/exposure for a total of 3 times)^6^, whereas Leinenga *et al*. conducted more frequent exposure in the whole animal brain (with the exposure frequency of 7 days/exposure for a total of 7 times)^7^. It is evident that the plaque reduction efficiency highly correlates with the BBB-opened area and exposure frequency, as hemisphere opening induced a ~20% plaque reduction^6^ whereas whole brain exposure induced ~75% plaque reduction^7^. A potential direction for improving the plaque reduction efficiency for our future investigation is to increase the BBB-opened area by increasing the exposure spot to either cover the single hemisphere or the whole brain. The issue of safety with multiple exposures has been discussed previously, showing that repeated FUS treatments did not pose additional hazard to the brain tissue in healthy brains^23–25^.

Neuroimaging biomarkers in Alzheimer’s disease have already been widely reported; for example, beta amyloid and tau protein burden can be obtained from nuclear imaging such as positron emission tomography (PET) and single-positron-emission computed tomography (SPECT)^26^. *In vivo* neural molecular imaging has played an extremely important role not only in diagnosis, but also in longitudinal characterization of disease progression for Alzheimer’s patients. To date, the efficient development of Alzheimer’s drugs requires quantitative evaluation by non-invasive *in vivo* neuroimaging. Currently, radiolabeled anti-Aβ compounds are prospective tools for further differential diagnosis between amyloid positive and negative forms of dementia, and can serve as a prognostic tool in patients with mild cognitive impairment (MCI)^27^.

To achieve the early diagnosis of Alzheimer’s, several *in vivo* PET radio-ligands have been developed and applied in preclinical and clinical trials, such as the recently developed tau protein ligands ^18^F-T807 and ^18^F-T808^28,29^. AV-45 is one of the potential tracers that can perform early AD diagnosis due to its high specificity and sensitivity in Aβ plague detection^30,31^. In addition, Tau species are considered promising biomarkers for early phase Alzheimer’s^32^. Yet, most of the Aβ PET (such as AV-45) or Tau detection fails to produce a full neurodegenerative map due to the limited penetration of the tracers across the BBB. Other under-developed PET antibody-based ligands also have the same hurdle in penetrating the BBB^33^. In addition to the therapeutic applications of FUS-induced BBB opening, it can also be used to enhance the delivery of PET tracers into the brain (including antibody-like, carbohydrate, or glycolipids tracers) to enhance neuritic plaque/neurofibrillary tangle binding, which will provide improvements in the detection sensitivity and specificity for early diagnosis of Alzheimer’s disease.

Preclinical studies (this study and other published reports) have shown that significant plaque reduction caused by FUS-BBB opening can be achieved and some studies have demonstrated associated improvement in behavior. However, it should be noted that, although a number of clinical studies have confirmed a positive correlation between Aβ-plaque reduction and behavior amelioration^6,7^, there is still a report showing that Aβ-plaque reduction may not necessarily lead to improved patient outcomes^34^. Of note, unlike previous studies showing Aβ-plaque reduction in response to GSK-3, it has been previously reported that GSK-3 inhibition can also interfere with neurofibrillary tangle formation^35,36^. Therefore, the proposed GSK-3 inhibitor-enhanced delivery via focused ultrasound might improve the therapeutic outcome for Alzheimer’s disease.

## Methods

### Animals and experimental design

All animal experiments conducted in this study were approved by the Institutional Animal Care and Use Committee (IACUC), Chang Gung University and we adhered to their experimental animal care guidelines (protocol number: CGU11-098). 24 APPswe/PSEN1-dE9 transgenic, aged from 12–14 months old) and 2 Wild-type (C57BL/6) male mice were employed (bred from the Department of Physiology, Taipei medical university, Taiwan). In addition, 9 normal ICR mice were acquired from an AAALAC-qualified animal center (BioLASCO, Co., Ltd., Taiwan) for FUS system confirmation and all animals were then housed at Chang Gung University. The animals were divided into two groups. In group 1, the aim was to optimize the FUS exposure parameter via the evaluation of Gd-DTPA (Magnevist^®^, Bayer HealthCare, NJ, USA) permeability via contrast-enhanced MRI *in vivo* monitoring and Evans blue (Sigma, St. Louis, MO, USA) penetration via brain sectional observation in normal mice (ICR) (n = 9; see Supplementary Fig. S2). In group 2, GSK-3 inhibitor (AR-A014418) were administered intraperitoneal (IP) in the dose of 21 μmol/kg body weight and FUS exposure was conducted weekly (for five weeks). The animals were divided into 4 sub-groups: (1) Without any treatment (denoted as Control, n = 3), (2) FUS-induced BBB opening alone (denoted as FUS; n = 6), (3) GSK-3 inhibitor administration alone (denoted as AR; n = 6), and (4) Combined FUS-induced BBB opening with GSK-3 inhibitor administration (denoted as AR + FUS; n = 9). The GSK-3 inhibitor AR-A014418 was employed in this study (Sigma, St. Louis, MO, USA; molecular weight = 308 Da), which is a thiazole, N-(4-methoxybenzyl)-N’-(5-nitro-1, 3-thiazol-2-yl) urea that has been shown to be a selective and potent inhibitor of GSK-3^37,38^.

### Focused ultrasound exposure

A FUS transducer (Imasonic, France; diameter = 60 mm, radius of curvature = 80 mm, frequency = 400 kHz) was applied to generate concentrated ultrasound energy. An arbitrary function generator (Agilent, Palo Alto, CA, USA) was used to produce the driving signal, which was fed to a radio frequency power amplifier (Advanced Surgical Systems, Tucson, AZ, USA) operating in burst mode. Anesthetized animals were immobilized on a stereotactic frame and a PE-10 catheter was inserted into the tail vein. SonoVue^®^ SF6-filled ultrasound microbubbles (2–5 µm, 10 µl/mouse; Bracco, Milan, Italy) were administered intravenously before treatment. The right hippocampus (see Supplementary Fig. S1) was then exposed to burst-mode ultrasound (acoustic pressure = 0, 0.41, 0.5 MPa for group 1 and 0.41 MPa for group 2; burst length = 10 ms; pulse repetition frequency = 1 Hz; exposure time = 60 s). After FUS exposure, the group-1 animals obtained contrast-enhanced MRI immediately after a bolus injection of Gd-DTPA and Evans blue dye administration to identify BBB-opened region (details see the Supplementary methods and Fig. S2A).

In group 2, the animals were sub-grouped to four, with the GSK-3 inhibitor administration involved animals (AR and AR + FUS group) were administered intraperitoneal (IP) injections of GSK-3 inhibitor (21 μmol/kg, AR-A014418, MW = 308.3, Sigma, St. Louis, MO, USA) weekly and dissolved in 1% dimethyl sulfoxide (DMSO)^38^. In sub-group which involved FUS-BBB opening (FUS and AR + FUS group), FUS-BBB opening was conducted via 0.41-MPa exposure level. The mice were sacrificed after 5-week FUS treatment. The brains of the mice were removed, frozen, and embedded in Optimal Compound Temperature compound (Sakura Finetek, USA). Embedded brains were sectioned serially into 10-μm-thick slices with a cryostat microtome (Leica, Germany). Brain samples were serially sectioned at the striatal region. A section thickness of 10 μm was used with the same coronal direction as in MR and PET imaging. The sections were separately stained with Thioflavin-S, GSK-3 antibody and examined by autoradiography (ARG).

### GSK-3 immunofluorescent staining

GSK-3 staining was employed to confirm suppression of GSK-3 activity. Frozen sectioned slides were subjected to immunofluorescence (IF) to confirm the distribution and determine the level of GSK-3 protein in the brain. Sections were fixed with 4% paraformaldehyde, rinsed with PBS, blocked with goat serum (Vector laboratories, CA, USA) for 30 min at room temperature, and incubated with mouse-anti-GSK-3α/β monoclonal antibody (SC-7291, 1:50, Santa Cruz, TX, USA) at 4 °C overnight. After rinsing with PBS, the sections were incubated with Cy3-conjugated goat-anti-mouse (115-165-003, 1:200, Jackson Immuno Research) for 1 hour at room temperature in the dark.

### Autoradiography (ARG)

ARG was also conducted to analyze the additive effect of FUS-BBB on plaque-reducing efficacy and to further confirm the *in vivo* AV-45 distribution measurement based on *in vivo* PET/CT imaging (Fig. S4). Brain sections were incubated with AV-45 radiotracer and attached to the phosphor imaging plates for 24 hours. ARG was performed to validate PET results. The method has been previously described in detail^39^. Briefly, a storage phosphor autoradiography plate (Fujifilm, BAS-MS2040, Fiji Photo Film, Japan) was exposed to the tissue slices overnight at −20 °C and read on the following day. The image plates were scanned by a FLA5100 (Fujifilm, Tokyo, Japan) with the resolution of 25 μm. The relative count intensity (LAU/mm^2^) of the sections in each image was quantified using Multi-Gauge version 3.0 software (Fujifilm, Tokyo, Japan). The regions of interest were placed at the bilateral cortex and hippocampus with/without sonication for comparison.

### Histological and Quantitative analysis

To demonstrate Aβ amyloid deposition in tissue sections, Thioflavin-S staining was conducted in this study. Brain sections were fixed with 4% paraformaldehyde, then washed with ethanol and incubated in filtered 1% Thioflavin-S. Quantitative analysis of the thioflavin-S and GSK-3 positive area was performed (ImageJ^®^) and interfaced with a digital CCD camera mounted on a fluorescent microscope (TissueGnostics, Austria). In the evaluation of thioflavin-S and GSK-3 distribution, due to the inconsistent selection of brain area for each slide, the distribution was presented as the ratio of fluorescent signal to total hippocampus area to show the objectiveness (as percentage (%)).

### Statistical analysis

All results are expressed as means ± standard error (SE) of duplicate or more measurements, obtained from three or more independent experiments. Data were analyzed using one-way ANOVA followed by post-ANOVA pair-wise tests using the Bonferroni correction (Prism 5, GraphPad Software Inc., CA, USA). A value of p < 0.05 was considered statistically significant.

## Electronic supplementary material

Supplementary Information
