# Supplementary material for: Focused Ultrasound-Induced Blood-Brain Barrier Opening Enhances GSK-3 Inhibitor Delivery for Amyloid-Beta Plaque Reduction
Source: Sci Rep. 2018 Aug 27;8:12882. doi: 10.1038/s41598-018-31071-8 (PMC6110796; doi:10.1038/s41598-018-31071-8)
Supplement: Supplementary file 1 — Supplementary Information [file 41598_2018_31071_MOESM1_ESM.pdf]

Focused Ultrasound Induced Blood-Brain Barrier Opening Enhances GSK-3 Inhibitor Delivery for Amyloid-Beta Plaque Reduction

Po-Hung Hsu[1,2], Ya-Tin Lin[3], Yi-Hsiu Chung[1], Kun-Ju Lin[4], Liang-Yo Yang[5,6], Tzu-Chen Yen[1,4], and Hao-Li Liu[2,7*]

[1]Center for Advanced Molecular Imaging and Translation, Chang Gung Memorial Hospital, Taoyuan, Taiwan
[2]Department of Electrical Engineering, Chang Gung University, Taoyuan, Taiwan
[3]Graduate Institute of Biomedical Sciences, Department of Physiology and Pharmacology, Chang Gung University, Taoyuan, Taiwan
[4]Department of Nuclear Medicine, Chang Gung Memorial Hospital, Taoyuan, Taiwan
[5]Department of Physiology, School of Medicine, College of Medicine, China Medical University, Taichung, Taiwan
[6]Department of Biotechnology, College of Medical and Health Science, Asia University, Taichung, Taiwan
[7]Department of Neurosurgery, Chang Gung Memorial Hospital, Taoyuan, Taiwan

***Address correspondence to:**
Hao-Li Liu, Ph.D.
Department of Electrical Engineering, Chang Gung University, 259 Wen-Hwa 1st Road, Taoyuan, 333, Taiwan.
Tel: +886-3-2118800 ext 5715. Fax: +886-3-2118026.
E-mail: haoliliu@mail.cgu.edu.tw

SUPPLEMENTARY METHODS

Focused ultrasound acoustic pressure measurement

A polyvinylidene fluoride (PVDF)-type hydrophone (Onda, Sunnyvale, CA, USA; calibration range: 50 kHz to 20 MHz) was used to measure the pressure distribution along the axis of the transducer and in the radial direction. Pressure was measured at low hydrophone output amplitudes and the plotted as Fig. S1C. The half-maximum pressure amplitude diameter of the produced focal spot were 2-3 mm, with the length was approximately 10 mm, respectively.

BBB-opened efficiency evaluated by Gd-DTPA and Evans blue

The BBB opening effect and its correlation with the molecular size is investigated by using gadolinium(Gd)-DTPA MR contrast agent (Magnevist®, Bayer HealthCare, NJ, USA, molecular weight = 938 Da) and Evans blue (EB, Sigma, St. Louis, MO, USA, molecular weight = 960 Da; form ~70 kDa EB-albumin complex after administering into blood circulation). Six wild-type C57B/6 male mouse were used in this evaluation, with three different acoustic pressures were tested to assess an optimal parameter to deliver GSK-3 inhibitor (0, 0.41, and 0.5 MPa; n=2 for each group). After FUS treatment, the animals were applied to MR imaging

immediately after a bolus injection of Magnevist® (1 mL/kg, intravenous) and EB (50 mg/kg, intravenous) to mark BBB-opened region, then were sacrificed 3 hours after Evans blue injection and the brain tissues were obtained for further evaluation (Fig. S2A). The brain tissues were extracted and then serially sectioned with the slice thickness of 1 mm. Sections nearby the hippocampus were acquired with a digital camera. Bilateral hippocampus areas were selected as region-of-interest (ROI) in MR images, with the image signal intensity (SI) been analyzed via ImageJ® to present the SI increase ratio of FUS treated lateral against contralateral.

Magnetic resonance imaging (MRI)

MRI images were acquired via a 7-Tesla magnetic resonance scanner (ClinScan, Bruker, Germany) and a 4-channel surface coil was placed on the top of the mouse brain. Contrast-enhanced MRI was used to assess increased BBB permeability via contrast agent administration. T1-weighted images were acquired with gradient echo FLASH sequence after a bolus injection of Magnevist® (1 mL/kg, intravenous injection); pulse repetition time (TR)/echo time (TE) = 300/3.81 msec; FOV = 21x25 mm^2; in-plane resolution = 173x256 pixels; slice thickness = 0.5 mm; flip angle =70°.

***In vivo* AV-45 PET/CT imaging**

To detect Aβ plaque reduction after the FUS treatment, the animals in three different treatment groups underwent brain FUS exposure and then *in vivo* ^{18}F-AV45 PET imaging (n = 6). The radiolabeled tracer was fully automated synthesis of ^{18}F-AV45 in our cGMP facility using Sumitomo modules with (E)-2-(2-(2-(5-(4-(tert-butoxycarbonyl(methyl)amino)styryl)pyridin-2-yloxy)ethoxy)ethoxy)ethyl 4-methylbenzenesulfonate (AV-105; Avid Radiopharmaceuticals, Philadelphia, PA, USA) as the precursor molecule. The product, ^{18}F-AV45, had a radiochemical purity of ＞90% and a specific activity of ＞36 TBq/mmol. The compound was radiochemically stable for at least 4 hours after completing the preparation[1].

The animals were given 22.83±2.32 MBq/0.2 cc of ^{18}F-AV45 (Eli Lily, IN, USA) via the tail vein with a catheter immediately after FUS sonication. According to the results of the pilot PET study, after 40 min of tracer injection, static brain images were acquired by microPET (Inveon, Siemens Medical Solutions, Germany). The animal preparation was performed as previously described. The protocol for static PET imaging was similar to that used for pilot PET studies which the energy window was set between 350 and 650 keV and the brains were placed in the center of the axial FOV to minimize scattered radiation. The PET images were reconstructed with the 3D-OSEM2D_z2 algorithm, and the total scanning time was 20 min to improve the overall image quality. A reference scan was performed immediately after the PET study with a microCT device (NanoSPECT/CT,

Mediso, Hungary). The image analysis was performed using PMOD (Technologies Ltd, Switzerland). The AV-45 PET images were co-registered with the microCT images and the MR mouse brain template of PMOD and manual ROI of hippocampus fused with co-registered AV-45 PET image, the AV-45 signal in the right and left hippocampus were determined, respectively[2].

Western immunoblot

All the samples were sonicated in 1% sodium dodecyl sulfate (SDS) and heated to 100 °C for 5 min. Equal amounts of proteins were separated by 10% SDS-polyacrylamide gel electrophoresis and transferred onto a polyvinylidene difluoride (PVDF) membrane (Millipore) using 200 mA for 70 min at 4 °C. After blocking with 5% non-fat milk for 1 hour, followed by incubation overnight with specific primary antibody, blots were incubated with the secondary antibody conjugated with HRP for 1 hour on the second day. The substrate solution (Western Lightning® plus ECL, PerkinElmer, Waltham, MA, USA) was added and reacted for 1 min and enhanced chemiluminescence signal was detected by ChemiDoc MP Imaging System with Image LabSoftware (Bio-Rad). Signals were normalized to GAPDH. The antibodies used were anti- GAPDH (1:2000, Genetex) and anti-pGSK3β (1:1000, Cell signaling).

SUPPLEMENTARY RESULTS

Determination of the FUS exposure level

After FUS-mediated BBB opening, animals were administered Magnevist® and EB intravenously, then T1-weighted MR images were acquired to evaluate the contrast signal intensity changes and EB marked BBB-opened regions. The result showed a similar signal intensity change (22.51±1.92% vs. 23.24±1.88%) between 0.41 MPa and 0.5 MPa group (Fig. S2B) but there was some obvious hemorrhages in 0.5 MPa group (Fig. S2C bottom panel). 0.41 MPa was therefore considered as a safe parameter to be employed in AD transgenic animal experiment.

Level of phosphorylated GSK-3β protein in transgenic mice hippocampus

The hippocampus of transgenic mice treated with AR alone and FUS +AR were collected, and the protein level of phosphorylated GSK-3β were further measured by western immunoblot. The phosphorylated GSK-3β were known as inactivated form of GSK-3β. The results showed that the combination of FUS and AR treatment significantly enhanced the inhibition effect of AR through the increase of phosphorylated GSK-3β (0.71±0.08 v.s. 1.5±0.27, $p<0.05$).

AV45-PET imaging to evaluate Aβ plaque reduction

We investigate the feasibility in using radiolabeled AV-45 PET imaging to detect the β amyloid depositions *in vivo.* Figure S4 compares the *in vivo* PET imaging observation of AV-45 uptake in the hippocampus in GSK-3 inhibitor alone (Fig. S4A) and GSK-3 inhibitor combined with FUS

exposure (Fig. S4B); the right hemisphere received FUS exposure and the left hemisphere served as the control). The fusion of AV-45 uptake distribution with MR image template is shown in Fig. S4C and Fig S4D, respectively, to demonstrate its anatomical distribution. Figure S3E showed the corresponding EB distribution for reference of the BBB-opened area. For GSK-3 inhibitor alone, similar AV-45 uptake in both hemispheres of the hippocampus was observed (see Fig. S4A and S4C). After combining with sequential FUS treatment, the AV-45 uptake in the treated hippocampus was identified to be distribution changed from *in vivo* PET imaging observation, when comparing with the untreated hippocampus (see Fig. S4B and S4D). The ratio of radiological count between treated lateral to contralateral was shown in Fig. S4F. In AR alone group, the AV-45 uptake was similar in both hemisphere and the ratio was 1.01±0.01, whereas in AR+FUS group, the ratio was 0.97±0.02. Since the difference in signal change was not statistically significant ($p = 0.0781$; 40% in power analysis between means of 0.04 (1.01 vs 0.97)), a further high-sensitivity ex-vivo quantification examination, autoradiography (ARG), was further performed (demonstrated as the main result in Fig. 3).

SUPPLEMENTARY REFERENCE

1. Lin, K. J. et al. Whole-body biodistribution and brain PET imaging with [18F]AV-45, a novel amyloid imaging agent--a pilot study. Nucl Med Biol 37, 497-508, doi:10.1016/j.nucmedbio.2010.02.003 (2010).

2. Poisnel, G. et al. PET imaging with [18F]AV-45 in an APP/PS1-21 murine model of amyloid plaque deposition. Neurobiology of aging 33, 2561-2571, doi:10.1016/j.neurobiolaging.2011.12.024 (2012).

ADDITIONAL INFORMATION

Competing financial interests: The authors declare no competing financial interests.

SUPPLELENTARY FIGURES

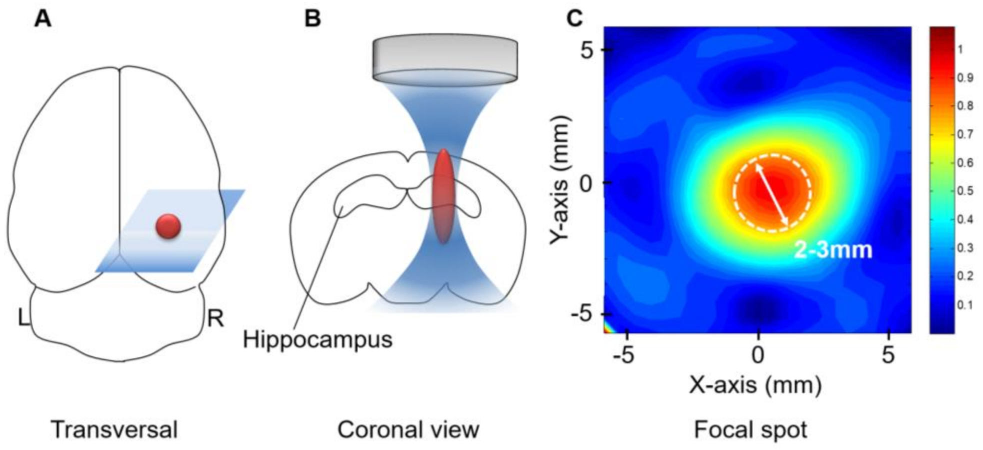

Figure S1. Focused ultrasound energy deposition. The location of hippocampus which was exposed to focused ultrasound in (A) transversal view and (B) coronal view, and (C) the measured pressure distribution on focused plane. The half-maximum pressure amplitude diameter of the produced focal spot were 2-3 mm, with the length was approximately 10 mm, respectively.

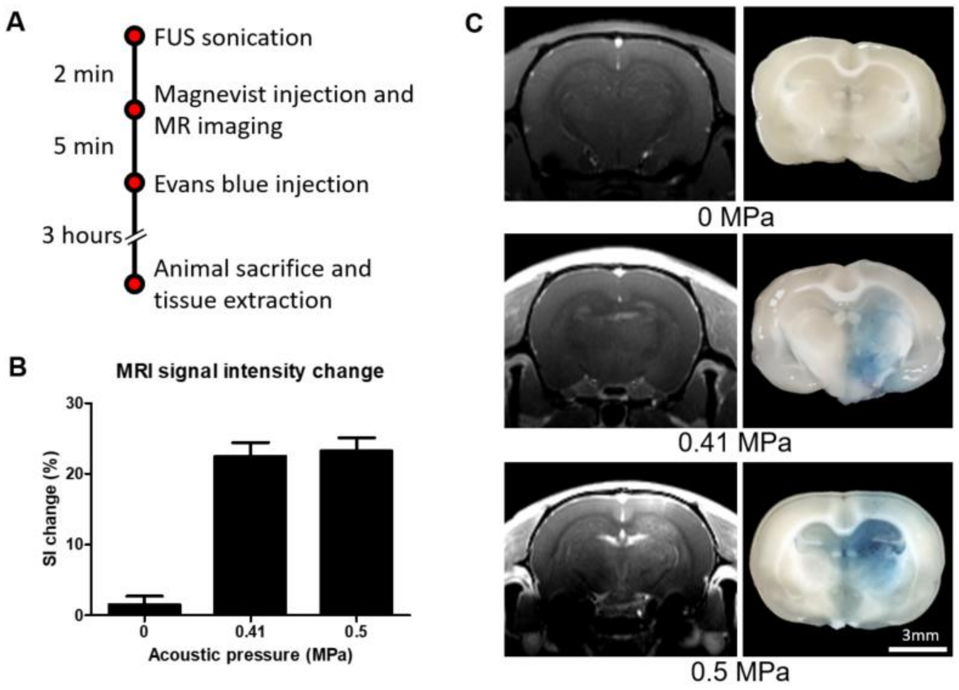

Figure S2. Contrast-enhanced MR signal intensity change and corresponding EB-stained brain observation for BBB-opening assessment. (A) Experimental procedure diagram showing from FUS exposure to animal tissue extraction; (B) MR signal intensity change (presented in the ratio of right to left hemisphere brain). Signal intensity showed a 22.51 ± 1.92% increase at 0.41-MPa exposure level, whereas with a similar 23.24 ± 1.88% at 0.5-MPa exposure level, comparing to a slight 1.54 ± 1.17% increase in sham control (i.e., 0-MPa group). (C) Gd-DTPA and Evans blue dye leakage marked BBB opening regions under 0- (n=3), 0.41- (n=3), and 0.5-MPa (n=3) exposure level.

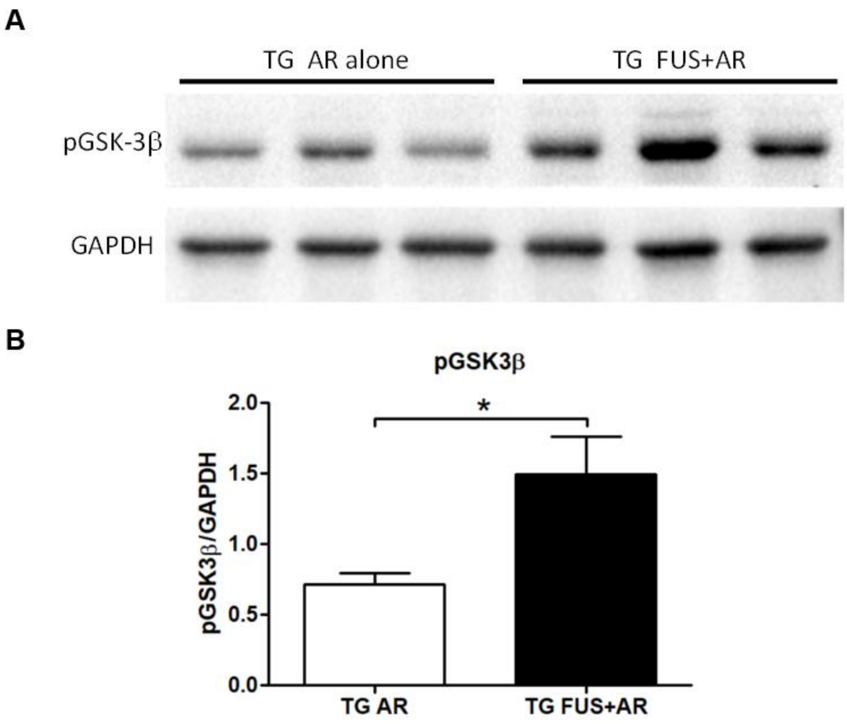

Figure S3. Level of phosphorylated GSK-3β protein in transgenic mice hippocampus The hippocampus of transgenic mice treated with AR alone or FUS+AR were collected, and the protein level of phosphorylated GSK-3β were further measured by western immunoblot. (A) The representative gel pictures were illustrated in triplicate. (B) The quantitative results showed that the combination of FUS and AR treatment significantly enhanced the inhibition effect of AR through the increase of phosphorylated GSK-3β (0.71±0.08 v.s. 1.5±0.27, $p<0.05$). AR = AR-A014418; FUS = focused ultrasound; TG = transgenic.

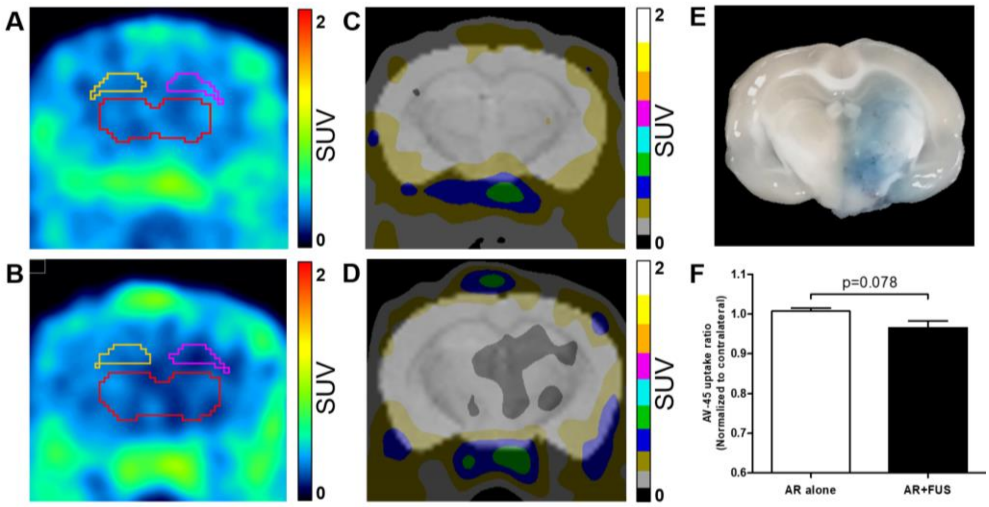

Figure S4. A representing image of AV-45 uptake in the transversal plane at the region of the hippocampus. (A) AR alone (n=6) and (B) AR+FUS treatment (n=6). (C-D) The AV-45 PET images fused with the MRI mouse brain template. The darker region represents the reduced AV-45 uptake after AR+FUS treatment. (E) A Evans blue dye was also applied to confirm the BBB-opened area in treated hemisphere in (D). (F) With 5 weeks of AR+FUS treatment, the AV-45 uptake slightly reduced at the FUS exposure site (by 3.96%) but without statistical significance (p = 0.078) compared to respective contralateral hippocampus (right/left). AR = AR-A014418; FUS = focused ultrasound; SUV = standardized uptake value.
